# ABO and RhD blood group are not associated with mortality and morbidity in critically ill patients; a multicentre observational study of 29 512 patients

**DOI:** 10.1186/s12871-022-01626-4

**Published:** 2022-04-02

**Authors:** Thomas Kander, Martin F. Bjurström, Attila Frigyesi, Magnus Jöud, Caroline U. Nilsson

**Affiliations:** 1 Anaesthesia & Intensive Care, Department of Clinical Sciences, Lund University, Skåne University Hospital, Entrégatan 7, 222 42 Lund, Sweden; 2 Clinical Immunology and Transfusion Medicine, Laboratory Medicine, Office for Medical Services, Region Skåne, Lund, Sweden

**Keywords:** ABO blood group, RhD blood group, Mortality, Morbidity, Blood group antigen, Critical care, Intensive care, Epidemiology

## Abstract

**Background:**

The ABO and RhD blood group represent antigens on the surface of erythrocytes. The ABO blood group antigens are also present on multiple other cells. Interestingly, previous studies have demonstrated associations between the blood group and many types of disease. The present study aimed to identifying associations between the ABO blood group, the RhD blood group, and morbidity and mortality in a mixed cohort and in six pre-defined subgroups of critically ill patients.

**Methods:**

Adult patients admitted to any of the five intensive care units (ICUs) in the Scania Region, Sweden, between February 2007 and April 2021 were eligible for inclusion. The outcomes were mortality analysed at 28– and 90–days as well as at the end of observation and morbidity measured using days alive and free of (DAF) invasive ventilation (DAF ventilation) and DAF circulatory support, including vasopressors or inotropes (DAF circulation), maximum Sequential Organ Failure Assessment score (SOFAmax) the first 28 days after admission and length of stay. All outcomes were analysed in separate multivariable regression models adjusted for age and sex. In addition, in a sensitivity analysis, five subgroups of patients with the main diagnoses sepsis, septic shock, acute respiratory distress syndrome, cardiac arrest and trauma were analysed using the same separate multivariable regression models.

**Results:**

In total, 29,512 unique patients were included in the analyses. There were no significant differences for any of the outcomes between non-O blood groups and blood group O, or between RhD blood groups. In the sensitivity analysis of subgroups, there were no differences in mortality between non-O blood groups and blood group O or between the RhD blood groups. AB was the most common blood group in the COVID-19 cohort.

**Conclusions:**

The ABO and RhD blood group do not influence mortality or morbidity in a general critically ill patient population.

**Supplementary Information:**

The online version contains supplementary material available at 10.1186/s12871-022-01626-4.

## Background

The ABO blood group represents genetic traits expressed as antigens on the surface of erythrocytes and multiple other cell types, including intestinal mucosa, endothelium, kidney, and heart [1, 2]. The RhD blood group is another major blood group, and the presence or absence of RhD antigen on the surface of erythrocytes (RhD blood group) is of clinical relevance [3]. Previous studies have demonstrated an association between the ABO blood group and several diseases [3]. For example, patients with non-O blood groups seem to be more susceptible to coronary artery disease and venous thrombosis than blood group O, and blood group A can influence the risk of infectious disease [3, 4]. Moreover, blood group O conveys a higher risk for bleeding complications, possibly due to observed lower circulating levels of von Willebrand factor (vWF) associated with this blood group [5]. Due to the emergence of the Coronavirus disease 2019 (COVID-19) pandemic, recent intense research efforts have been directed towards investigating the ABO blood group as putative risk factors for severe acute respiratory syndrome coronavirus-2 (SARS-CoV-2) disease. Although results are equivocal, blood group O appears to be protective against COVID-19 infection compared to non-O blood groups, particularly blood group A [6–8].

Links between the RhD blood group and health outcomes are unclear and have been far less studied in the critical care setting than the ABO blood group [3, 8].

Although many previous studies have demonstrated varying associations between the ABO blood group and outcomes in subgroups of critically ill patients [9–20], to our knowledge, only one previous original study has examined a mixed critically ill patient population [21]. In a register-based study (*n* = 7340), Slade et al. found that critically ill patients with blood group AB exhibited a higher 90-day survival probability compared to patients with non–AB blood groups (*p* = 0.049, unadjusted log-rank analysis) [21]. However, only 90-day mortality was investigated, and the analyses were not corrected for any confounding variables.

In an attempt to further investigate the influence of the ABO blood group on short-term, intermediate and long-term mortality and morbidity in a large cohort of mixed critically ill patients, we designed the current multicentre, register-based study. The primary aim was to identify any associations between the ABO blood group, RhD blood group, and morbidity and mortality in a main cohort and six pre-defined subgroups.

## Methods

### Study design and overview

The study was approved by the National Ethical Review Board, Lund, Sweden, with registration numbers 2014/916 and 2018/866. As the study was exclusively observational, the Board waived the requirement for informed consent. The manuscript was written following the STROBE statement for observational studies [22].

All patients ≥18 years at admittance to any of the five general ICUs in southern Sweden (two at a university hospital (Skåne University Hospital, Lund and Malmö) and three at county hospitals (Helsingborg, Ystad and Kristianstad) with a combined total of 35 beds, between February 2007 and April 2021, were included. The inclusion time was the time of admission to any of the participating ICUs. Tertiary paediatric, cardiothoracic and neurological patients were not included in this study as they received intensive care elsewhere. For patients with multiple ICU admissions, only the first admission was included. In addition, patients with an unknown ABO or RhD blood group or patients who were not critically ill were excluded. Examples of non-critically ill admitted patients include patients admitted after elective surgery and patients admitted due to a hospital shortage of general ward beds. Data were collected from the local quality register (PASIVA, Otimo Data AB, Kalmar, Sweden) and paired with data on ABO and RhD blood groups, extracted from the laboratory information system (Flexlab/DoReMi, Tieto Sweden AB, Stockholm, Sweden) at Clinical Immunology and Transfusion Medicine, Region Skåne, Sweden.

### Outcomes

Mortality was analysed at 28– and 90–days and at the end of observation (the point of data collection).

Morbidity was measured using days alive and free of (DAF) invasive ventilation (DAF ventilation) and DAF circulatory support, including vasopressors or inotropes (DAF circulation) the first 28 days after admission. As previously recommended, DAF was used without extra penalty for death [23]. Furthermore, the maximum Sequential Organ Failure Assessment–score (SOFA–max) during the ICU stay and the duration of the ICU stay itself (“Length of stay”) were used to assess morbidity.

### Statistics

The sample size was based on the number of available patients during the study period. Normality tests were used to test for normal distribution, and all continuous variables were found to be non-normally distributed and thus summarised with median (5–95 percentile) accordingly. Numbers were provided together with percentages.

All regression analyses were corrected for age and sex. All outcomes were analysed in separate multivariable regression models resulting in odds or hazard ratios for each ABO and RhD blood group using blood group O and RhD negative as references. Mortality at 28 (short-term) and 90 days (intermediate-term) after ICU admission were analysed using multivariable logistic regression. The Hosmer–Lemeshow goodness–of–fit test was used to ensure correct calibration of the logistic regression models. The Cox regression hazard assumption was tested before the Cox–regression analyses for long–term mortality.

As the distribution of the DAF, the length–of–stay, and the SOFA-max variables did not fit any commonly used regression analyses, these variables were recoded from continuous variables into categorical data. For the DAF variables, 24 h of treatment was used as cut off, i.e., DAF < 27 days = 1 and DAF ≥27 days = 0. For the length–of–stay and the SOFA-max variables, the cut-off was applied at 1.5 ICU–days and 7 points, respectively, which corresponded to the median of both variables. All *P*–values were two-tailed, and < 0.05 were considered significant. All analyses were performed using SPSS version 27 (SPSS Inc., Chicago, IL, USA).

### Sensitivity analyses

1) As previously described, ABO and RhD blood groups may affect the severity of illness [1, 3, 6, 14]. Hence, the primary analyses in the present study were not corrected for the severity of illness. However, in secondary sensitivity analyses, decided a priori, we tested if blood groups may add predictive value to the severity score Simplified Acute Physiology score 3 with estimated mortality risk (SAPS 3, EMR) by adding SAPS 3, EMR as an independent variable in the regression analyses.

2) Previous studies have demonstrated varying effects of the ABO and RhD blood group in different subgroups of critically ill patients [9–19]. To further evaluate these effects, we divided the main cohort into six subgroups with the main diagnoses: sepsis, septic shock, acute respiratory distress syndrome (ARDS), COVID-19, cardiac arrest and trauma. The primary multivariable regression analyses were planned to be repeated for each subgroup.

## Results

**Fig. 1:**
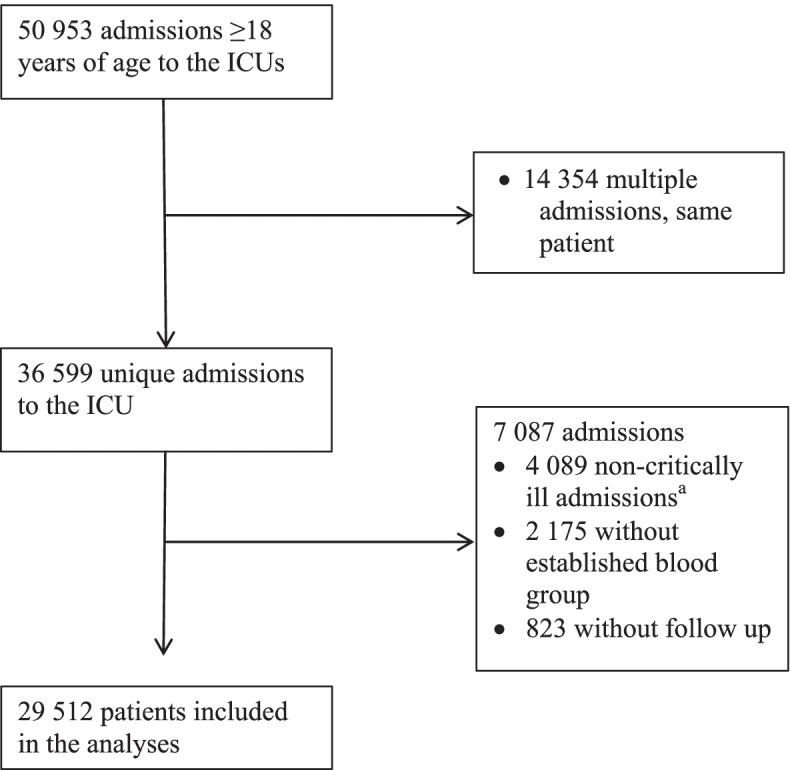
Consort diagram

In total, 29,512 unique patients were included in the analyses (Fig. 1). Detailed baseline characteristics stratified according to ABO and RhD blood group are shown in Table 1. In summary, baseline variables at admission were generally similar according to the ABO blood group, with the exceptions of age (*p* < 0.001), heart rate (*p* = 0.014) and leucocyte count (*p* = 0.031). There were no differences in baseline characteristics according to the RhD blood group. For the whole cohort, the median age was 67 years (25–85), 58% were men and the median SAPS 3 EMR, calibrated for Sweden in 2016, was 18% (1.0–73).

**Table 1:** Baseline characteristics^a^

ABO group	Total	A	B	AB	O	***P***-value^b^
**N**	**29,512**	**12,969**	**3421**	**1416**	**11,706**	
**ABO group current study, %**		44	12	4.7	40	N.A.
**ABO group in Sweden,%** [24]		45	11	5.0	39	N.A.
**Age, years**	67 (25–85)	67 (25–85)	66 (24–84)	66 (23–85)	68 (25–85)	<0.001
**Male sex**	16,956 (58)	7406 (57)	1194 (58)	802 (57)	6754 (58)	0.516
**SAPS 3 EMR,**^c^ **%**	18 (1.0–73)	18 (1.0–73)	18 (1.0–73)	18 (1.0–74)	19 (1.0–73)	0.762
**Comorbidities, %**						
Cancer^d^	9.0	9.2	8.5	8.1	9.0	0.591
Heart failure^e^	5.8	5.4	5.4	7.0	6.2	0.083
Blood malignancy	2.0	1.9	2.2	2.0	2.0	0.858
Cirrhosis	2.0	1.9	2.1	2.0	1.8	0.803
Immunosuppression^f^	4.9	5.1	4.6	4.2	4.7	0.569
**Admission route, %**						
Emergency dept.	44	44	43	44	45	0.803
Surgical theatre	11	11	12	12	11	0.757
General ward	30	31	30	30	30	0.823
Other ICU	3.7	3.7	4.0	3.1	3.6	0.110
PACU^g^	3.6	3.6	3.5	3.3	3.8	0.858
Missing data	6.8	6.8	7.3	7.0	6.8	0.686
**Reason for admission**^h^**, %**						
Sepsis^i^	10	10	9.4	11	10	0.371
Septic shock	4.6	4.8	3.9	4.2	4.7	0.097
ARDS	19	19	19	19	19	0.757
Covid–19	1.1	1.2	1.3	1.3	1.0	0.118
Cardiac arrest	8.7	8.9	8.9	8.7	8.3	0.371
Cardiovascular^j^	30	30	29	31	30	0.722
Trauma	5.4	5.4	5.5	4.7	5.4	0.664
Hepatic	5.2	5.2	5.4	4.8	5.1	0.819
Abdominal^k^	23	23	23	22	23	0.849
CNS^l^	39	39	40	41	38	0.268
Renal	16	16	16	17	17	0.646
Metabolic	17	17	17	16	17	0.687
**Days in hospital before admission**	0 (0–11)	0 (0–12)	0 (0–12)	0 (0–11)	0 (0–11)	0.675
**Physiological parameters**^m^						
Heart rate /min	100 (60–150)	100 (59–147)	100 (60–149)	100 (60–150)	100 (61–150)	0.014
SBP^n^, mmHg	100 (52–162)	100 (54–160)	100 (50–165)	100 (55–163)	100 (50–164)	0.755
Body temp., °C	37.0 (34.8–39.0)	37.0 (34.8–39.0)	37.0 (35.0–39.0)	37.0 (34.8–39.0)	37.0 (34.8–39.0)	0.658
Leucocytes, x10^9^/L	12 (4.0–28)	12 (3.8–27)	12 (4.1–28)	13 (4.0–29)	13 (4.1–28)	0.031
Creatinine, μmol/L	94 (46–392)	94 (46–381)	94 (47–399)	92 (47–399)	95 (46–399)	0.604
Bilirubin, μmol/L	10 (3.0–46)	10 (3.0–45)	10 (3.0–50)	10 (3.0–49)	10 (3.0–46)	0.241
Platelet count, x10^9^/L	211 (68–427)	212 (66–427)	208 (67–431)	213 (72–435)	211 (70–424)	0.368
Blood pH	7.34 (7.01–7.49)	7.34 (7.01–7.49)	7.34 (7.01–7.48)	7.34 (7.03–7.49)	7.34 (7.01–7.49)	0.650

^a^Continuous variables are presented as median (5–95 percentile) and numbers with %

^b^Chi-square test or Mann-Whitney-U test for differences between RhD status groups

^c^Estimated mortality risk calibrated for Swedish conditions

^d^Spread beyond regional lymph nodes

^e^Chronic heart failure NYHA IV

^f^Chronic steroid treatment corresponding to ≥0.3 mg/kg prednisolone/day,

radiation or chemotherapy

^g^Post-Anesthesia Care Unit

^h^Patients may have multiple reasons for admission

^i^According to Sepsis 2 definition

^j^Hypovolemia, cardiac shock, mixed shock, anaphylactic shock or arrhythmia

^k^Gastrointestinal bleeding, acute abdomen or pancreatitis

^l^Convulsions, decreased consciousness, coma, delirium or intracranial volume effect

^m^Registered ± 90 min from admission

^n^Systolic Blood Pressure

No imputations of missing data were performed. All baseline variables had <10% missing data.

### Outcomes

Descriptive values for the outcomes are summarised in Table 2. There were no differences according to the ABO or the RhD blood group in these analyses.

**Table 2:** Outcomes^a^

Blood group	Total	A	B	AB	O	***P***-value^b^	RhD positive	RhD negative	***P***-value^c^
**N**	**29,512**	**12,969**	**3421**	**1416**	**11,706**		**24,797**	**4715**	
28–day mortality, %	24	24	23	25	25	0.448	25	24	0.451
90–day mortality, %	29	29	28	30	29	0.237	29	29	0.626
DAFvent^d^	27 (0–28)	27 (16–28)	27 (19–28)	27 (17–28)	27 (16–28)	0.774	27 (0–28)	27 (0–28)	0.188
DAFcirc^e^	28 (0–28)	28 (0–28)	28 (0–28)	28 (0–28)	28 (0–28)	0.356	28 (0–28)	28 (0–28)	0.608
SOFA-max^f^	7 (1–15)	7 (1–15)	7 (1–16)	7 (1–15)	7 (1–15)	0.867	7 (1–11)	7 (1–11)	0.813
Length-of-stay, days	1.5 (0.2–12)	1.5 (0.2–12)	1.5 (0.2–12)	1.6 (0.2–11)	1.5 (0.2–12)	0.154	1.5 (0.7–3.4)	1.5 (0.7–3.3)	0.646

^a^Continuous variables are presented as median (5–95 percentile) and numbers with (%)

^b^Chi-square test or Kruskal-Wallis test for differences between ABO blood groups

^c^Chi-square test or Mann-Whitney-U test for differences between RhD status groups

^d^Days alive and free of invasive ventilation

^e^Days alive and free of circulatory support

^f^Maximum of Sequential Organ Failure Assessment score during the ICU stay

Detailed results of the multivariable analyses are presented in Table 3. The goodness of fit test was not significant for any of the models. The proportional hazards assumption was not violated for any of the models (*P* > 0.05). In summary, there were no differences for any of the outcomes between non-O blood groups and blood group O, or between RhD blood groups. In the first set of sensitivity analyses, including SAPS 3 as an independent variable, there were no differences in any of the mortality/morbidity outcomes between non-O blood groups and blood group O, or between RhD blood groups (Table 4). SAPS 3 was strongly associated with all outcomes. In the second set of sensitivity analyses, where the main cohort was divided into six pre-defined subgroups, the lack of associations between the mortality/morbidity outcomes and ABO blood group and RhD blood group remained largely unchanged in five of the subgroups as compared to the main analyses. The COVID-19 cohort was not analysed, given the low number of patients. Nevertheless, in the subgroups septic shock and cardiac arrest, blood group AB was associated with a longer length-of-stay than blood group O. In the ARDS-subgroup, blood group A was associated with DAF ventilation. There were no differences in mortality variables between non-O blood groups and blood group O, or between RhD blood group, in any subgroups. Further details are provided in Additional file 1.

**Table 3:** Multivariable regression analyses with 95% confidence interval for odds or hazard ratio^a^

Blood group	A	B	AB	RhD positive
**Mortality**				
28–day mortality	0.94–1.06	0.90–1.09	0.94–1.22	0.97–1.13
90–day mortality	0.95–1.07	0.90–1.08	0.97–1.25	0.96–1.11
Long-term mortality^b^	0.99–1.06	0.94–1.05	0.98–1.14	0.98–1.08
**Morbidity**				
DAFvent^c^	0.99–1.09	0.95–1.11	0.94–1.18	1.00–1.14^d^
DAFcirc^e^	0.93–1.03	0.96–1.13	0.98–1.24	0.97–1.10
SOFA-max^f^	0.95–1.06	0.96–1.13	0.93–1.19	0.96–1.10
Length-of-stay	0.95–1.05	0.98–1.14	0.97–1.21	0.96–1.08

^a^All analyses were corrected for age and sex. All outcomes were analysed in separate multivariable regression models resulting in odds or hazard ratio for each ABO blood group and RhD status using blood group O and RhD negative as references

^b^Cox regression

^c^Days alive and free of invasive ventilation

^d^Non-significant, *P* = 0.052

^e^Days alive and free of circulatory support

^f^Maximum of Sequential Organ Failure Assessment score during the ICU stay

**Table 4:** First set of sensitivity analyses. Multivariable regression analyses with 95% confidence interval for odds or hazard ratio^a^

	SAPS3, EMR^b^	ABO or RhD blood group			
		A	B	AB	RhD positive
**Mortality**					
28–day mortality	1.05–1.05*	0.93–1.07	0.87–1.08	0.91–1.23	0.94–1.11
90–day mortality	1.04–1.05*	0.95–1.08	0.88–1.07	0.94–1.25	0.93–1.10
Long-term mortality^c^	1.02–1.02*	0.99–1.06	0.93–1.04	0.94–1.10	0.96–1.05
**Morbidity**					
DAFvent^d^	1.05–1.05*	0.99–1.12	0.94–1.13	0.93–1.21	0.97–1.13
DAFcirc^e^	1.04–1.04*	0.92–1.03	0.95–1.15	0.97–1.27	0.93–1.08
SOFA-max^f^	1.06–1.07*	0.95–1.09	0.94–1.15	0.93–1.24	0.91–1.07
Length-of-stay	1.01–1.01*	0.96–1.06	0.98–1.15	0.97–1.23	0.96–1.09

^a^All analyses were corrected for age and sex. All outcomes were analysed in separate multivariable regression models resulting in odds or hazard ratio for each ABO blood group and RhD status using blood group O and RhD negative as references

^b^Simplified Acute Physiology Score 3 with estimated mortality risk

^c^Cox regression

^d^Days alive and free of invasive ventilation

^e^Days alive and free of circulatory support

^f^Maximum of Sequential Organ Failure Assessment score during the ICU stay

* *P* < 0.05

The distribution of COVID-19 patients on the ABO and RhD blood group demonstrated that blood group AB was more common in the COVID-19 group than the non-COVID-19 group (Additional file 2).

## Discussion

In this large multicentre observational study, we have demonstrated that neither the ABO nor the RhD blood group is associated with mortality or morbidity in a mixed critically ill patient population. In sensitivity analyses, the ABO and the RhD blood group did not add predictive value to the SAPS 3. There were no differences in mortality in five different subgroups between non-O blood groups and blood group O or between the RhD blood groups. Furthermore, we could not demonstrate any differences in the number of transfused units according to the ABO or the RhD blood group.

We did not correct for multiple testing as this would increase the risk of not detecting small differences according to ABO or RhD blood group (statistical type 2 error). It should also be noted that the differences in the baseline variables age, heart rate and leucocyte count according to the ABO blood group may be a side effect of this and thus represent statistical type 1 errors.

Based on previous findings, the ABO blood group is likely to affect homeostasis through several mechanisms [1–3]. However, our results imply that this effect is subtle, if not irrelevant, in a large mixed cohort of critically ill patients. In addition, the ABO blood group may influence outcomes in certain clinical populations suffering from specific diseases; nevertheless, in contrast to some previous reports, we did not observe a mortality effect in any of the subgroups (sepsis, septic shock, ARDS, cardiac arrest and trauma). However, it should be noted that in the subgroups septic shock and cardiac arrest, blood group AB was associated with a longer length-of-stay as compared to blood group O. In the ARDS-subgroup, blood group A was associated with increased ventilator time as measured with DAF ventilation.

In contrast to the absence of associations between the ABO blood group and outcomes in the present study of mixed critically ill patients, Slade et al. found that blood group AB conferred a 90-day mortality benefit compared to other ABO blood groups in a similar but smaller patient cohort in the United Kingdom [21]. However, it should be noted that only 3% of patients had blood group AB. Although baseline data were similar according to the ABO blood group, the mortality analyses were unadjusted, which may at least in part explain the differences compared to our results.

In the present study, patients with blood group AB demonstrated a higher susceptibility to severe COVID-19 disease. However, we only analysed 338 patients with COVID-19 compared to 29,174 patients without COVID-19, so these results should be interpreted carefully. Although, the results are in agreement with some previous findings, most studies indicate that blood group A is associated with severe COVID-19 and blood group O might be protective against the disease [6].

The influence of the ABO-blood group has also been investigated in critically ill patients with non-COVID-19 diseases, such as sepsis and ARDS. In two separate studies, Reilly et al. demonstrated that blood group A is associated with increased risk for ARDS in sepsis and trauma, possibly due to dysfunction of endothelium and microvasculature, as indicated by altered levels of biomarkers (e.g., soluble thrombomodulin and selectins) [14, 15]. These findings are supported by results from a large study of critically ill sepsis patients evaluating the ABO blood group and biomarkers of endothelial damage, which demonstrated moderately decreased mortality for blood group B [12]. In the present study, the potential effect of the ABO-blood group did not influence morbidity and mortality outcomes for any of the sepsis (*n* = 3016), septic shock (*n* = 1366) or ARDS (*n* = 5642) subgroups.

The increased risk of bleeding for patients with blood group O is mainly mediated by lower vWF levels (and associated lower factor VIII levels) [5]. Blood group O has been associated with worse outcomes in severe trauma and increased transfusion volume in severe abdominal trauma, which could be due to the proposed haemostatic effects [17, 18]. As mentioned above, Reilly et al. found a connection between blood group A and ARDS risk in trauma patients [14, 15]. Here, congruent with findings from two previous studies, we did not observe worse outcomes for blood group O in the trauma subgroup [9, 10].

The RhD blood group is less studied than the ABO system for its association with diseases, including critical illness. We found no effect related to the RhD blood group on mortality or morbidity in the general critically ill population or any specific subgroup. The RhD antigen is only expressed on red blood cells. Therefore, it is primarily of clinical interest in the context of haemolytic reactions (of the newborn and in the case of blood transfusion). Individuals who are RhD-negative may have some protection against COVID-19 infection, but the pathophysiological basis is unclear [8].

Although an individual cannot change ABO or RhD blood group, except in the rare case of stem cell transplantation, the results of the present study expand important knowledge regarding associations between the ABO – RhD blood groups and mortality – morbidity. Therefore, besides epidemiological studies, such as the present work, studies investigating pathophysiological alterations associated with blood groups, e.g., changes in vWF levels, blockage of virus-receptors and endotheliopathy, should be given high priority since detailed mechanistic insights may potentially guide the development of future personalised therapies.

### Limitations and strengths

We recognise the limitations in the present study given its retrospective nature, including the absence of a published study protocol. Secondly, data on ethnicity is missing. Thirdly, the division into subgroups in the second set of sensitivity analyses was performed based on diagnoses coded by the treating physician. Hence it cannot be ruled out that some diagnoses were missed. Although the selection of subgroups was performed based on previously demonstrated associations between the ABO blood group and outcomes [9–19], it can be argued that different selections of subgroups would have yielded a different result. It should also be noted that overlap between the subgroups may have introduced a bias in the second set of sensitivity analyses. Fourthly, the COVID-19 cohort was deemed too small for robust analyses of independent risk factors. Finally, no correction for multiple testing was performed as this would increase the risk of not identifying true differences. Strengths of the study include a larger sample size compared to previous similar studies, comprehensive datasets and the multicentre design.

## Conclusions

We have demonstrated that neither the ABO nor the RhD blood group is associated with mortality or morbidity in a mixed critically ill patient population. In sensitivity analyses, the ABO and RhD blood group did not add predictive value to the SAPS 3. There were no differences in mortality in five different subgroup analyses between non-O blood groups and blood group O or between RhD blood groups.

## Supplementary Information

**Additional file 1.**

**Additional file 2.**

## Data Availability

The datasets used and/or analysed during the current study are available from the corresponding author on reasonable request.

## Abbreviations

ARDS: Acute respiratory distress syndrome
COVID-19: Coronavirus disease 2019
DAF: Days alive and free of
ICU: Intensive care unit
SAPS 3: Simplified acute physiology score 3
SARS-CoV-2: Severe acute respiratory syndrome coronavirus-2
SOFA: Sequential organ failure assessment score
vWF: Von willebrand factor
