# Supplementary material for: ABO and RhD blood group are not associated with mortality and morbidity in critically ill patients; a multicentre observational study of 29 512 patients
Source: BMC Anesthesiol. 2022 Apr 2;22:91. doi: 10.1186/s12871-022-01626-4 (PMC8976170; doi:10.1186/s12871-022-01626-4)
Supplement: Supplementary file 1 — Additional file 1. [file 12871_2022_1626_MOESM1_ESM.docx]

**Additional file 1. Outcomes for pre-defined subgroups**

| **Subgroup Sepsis, n= 3 016. Multivariable regression analyses with 95% confidence interval for odds or hazard ratio^^[[1]](#endnote-1)^^** | | | | | |
| --- | --- | --- | --- | --- | --- |
|  | **Blgrp A** | **Blgrp B** | **Blgrp AB** | **RhD positive** |  |
| **Mortality** |  |  |  |  |  |
| 28–day mortality | 0.913 – 1.278 | 0.765 – 1.309 | 0.717 – 1.499 | 0.975 – 1.496 |  |
| 90–day mortality | 0.963 – 1.332 | 0.819 – 1.369 | 0.868 – 1.753 | 0.901 – 1.350 |  |
| Long-term mortality^[[2]](#endnote-2)^ | 0.961 – 1.175 | 0.971 – 1.327 | 0.850 – 1.328 | 0.932 – 1.199 |  |
| **Morbidity** |  |  |  |  |  |
| DAFvent^^[[3]](#endnote-3)^^ | 0.927 – 1.273 | 0.787 – 1.295 | 0.744 – 1.478 | 0.860 – 1.273 |  |
| DAFcirc^^[[4]](#endnote-4)^^ | 0.860 – 1.187 | 0.767 – 1.272 | 0.748 – 1.513 | 0.871 – 1.296 |  |
| SOFA-max^^[[5]](#endnote-5)^^ | 0.841 – 1.235 | 0.795 – 1.473 | 0.619 – 1.414 | 0.861 – 1.382 |  |
| Length-of-stay | 0.842 – 1.169 | 0.858 – 1.455 | 0.924 – 1.961 | 0.899 – 1.349 |  |

| **Subgroup Septic shock, n= 1 366. Multivariable regression analyses with 95% confidence interval for odds or hazard ratio^^[[6]](#endnote-6)^^** | | | | | |
| --- | --- | --- | --- | --- | --- |
|  | **Blgrp A** | **Blgrp B** | **Blgrp AB** | **RhD positive** |  |
| **Mortality** |  |  |  |  |  |
| 28–day mortality | 0.861 – 1.400 | 0.777 – 1.735 | 0.390 – 1.295 | 0.771 – 1.415 |  |
| 90–day mortality | 0.931 – 1.490 | 0.664 – 1.457 | 0.467 – 1.430 | 0.699 – 1.251 |  |
| Long-term mortality^[[7]](#endnote-7)^ | 0.938 – 1.281 | 0.803 – 1.363 | 0.624 – 1.309 | 0.811 – 1.195 |  |
| **Morbidity** |  |  |  |  |  |
| DAFvent^^[[8]](#endnote-8)^^ | 0.941 – 1.508 | 0.837 – 1.846 | 0.543 – 1.594 | 0.870 – 1.553 |  |
| DAFcirc^^[[9]](#endnote-9)^^ | 0.755 – 1.322 | 0.792 – 2.116 | 0.765 – 3.374 | 0.755 – 1.507 |  |
| SOFA-max^^[[10]](#endnote-10)^^ | 0.709 – 1.445 | 0.524 – 1.662 | 0.411 – 2.205 | 0.772 – 1.807 |  |
| Length-of-stay | 0.783 – 1.285 | 0.708 – 1.630 | 1.136 – 4.642* | 0.837 – 1.542 |  |

| **Subgroup ARDS, n=5 642. Multivariable regression analyses with 95% confidence interval for odds or hazard ratio^^[[11]](#endnote-11)^^** | | | | | |
| --- | --- | --- | --- | --- | --- |
|  | **Blgrp A** | **Blgrp B** | **Blgrp AB** | **RhD positive** |  |
| **Mortality** |  |  |  |  |  |
| 28–day mortality | 0.924 – 1.186 | 0.941 – 1.378 | 0.763 – 1.328 | 0.993 – 1.361 |  |
| 90–day mortality | 0.902 – 1.148 | 0.837 – 1.213 | 0.736 – 1.256 | 0.937 – 1.266 |  |
| Long-term mortality^[[12]](#endnote-12)^ | 0.985 – 1.132 | 0.975 – 1.207 | 0.890 – 1.216 | 0.985 – 1.172 |  |
| **Morbidity** |  |  |  |  |  |
| DAFvent^^[[13]](#endnote-13)^^ | 1.014 – 1.283* | 0.867 – 1.242 | 0.856 – 1.443 | 0.926 – 1.240 |  |
| DAFcirc^^[[14]](#endnote-14)^^ | 0.888 – 1.119 | 0.866 – 1.237 | 0.722 – 1.205 | 0.871 – 1.162 |  |
| SOFA-max^^[[15]](#endnote-15)^^ | 0.930 – 1.185 | 0.803 – 1.162 | 0.748 – 1.270 | 0.873 – 1.181 |  |
| Length-of-stay | 0.867 – 1.105 | 0.762 – 1.102 | 0.659 – 1.113 | 0.828 – 1.120 |  |

| **Subgroup Covid-19, n=338. Multivariable regression analyses were not performed due to the low number of patients** |
| --- |

| **Subgroup Cardiac arrest, n=2 562. Multivariable regression analyses with 95% confidence interval for odds or hazard ratio^^[[16]](#endnote-16)^^** | | | | | |
| --- | --- | --- | --- | --- | --- |
|  | **Blgrp A** | **Blgrp B** | **Blgrp AB** | **RhD positive** |  |
| **Mortality** |  |  |  |  |  |
| 28–day mortality | 0.763 – 1.085 | 0.616 – 1.041 | 0.585 – 1.261 | 0.725 – 1.117 |  |
| 90–day mortality | 0.810 – 1.160 | 0.624 – 1.062 | 0.591 – 1.285 | 0.742 – 1.151 |  |
| Long-term mortality^[[17]](#endnote-17)^ | 0.899 – 1.098 | 0.756 – 1.029 | 0.695 – 1.096 | 0.807 – 1.029 |  |
| **Morbidity** |  |  |  |  |  |
| DAFvent^^[[18]](#endnote-18)^^ | 0.854 – 1.350 | 0.688 – 1.353 | 0.607 – 1.647 | 0.798 – 1.389 |  |
| DAFcirc^^[[19]](#endnote-19)^^ | 0.810 – 1.202 | 0.684 – 1.228 | 0.599 – 1.399 | 0.802 – 1.295 |  |
| SOFA-max^^[[20]](#endnote-20)^^ | 0.955 – 1.501 | 0.811 – 1.600 | 0.819 – 2.360 | 0.634 – 1.130 |  |
| Length-of-stay | 0.921 – 1.305 | 0.959 – 1.633 | 1.111 – 2.506* | 0.840 – 1.289 |  |

| **Subgroup Trauma, n=1 583. Multivariable regression analyses with 95% confidence interval for odds or hazard ratio^^[[21]](#endnote-21)^^** | | | | | |
| --- | --- | --- | --- | --- | --- |
|  | **Blgrp A** | **Blgrp B** | **Blgrp AB** | **RhD positive** |  |
| **Mortality** |  |  |  |  |  |
| 28–day mortality | 0.750 – 1.551 | 0.528 – 1.530 | 0.270 – 1.895 | 0.633 – 1.670 |  |
| 90–day mortality | 0.691 – 1.364 | 0.671 – 1.907 | 0.202 – 1.420 | 0.729 – 1.836 |  |
| Long-term mortality^[[22]](#endnote-22)^ | 0.838 – 1.335 | 0.733 – 1.536 | 0.339 – 1.229 | 0.746 – 1.362 |  |
| **Morbidity** |  |  |  |  |  |
| DAFvent^^[[23]](#endnote-23)^^ | 0.795 – 1.251 | 0.619 – 1.242 | 0.465 – 1.362 | 0.746 – 1.361 |  |
| DAFcirc^^[[24]](#endnote-24)^^ | 0.671 – 1.144 | 0.837 – 1.833 | 0.449 – 1.629 | 0.705 – 1.416 |  |
| SOFA-max^^[[25]](#endnote-25)^^ | 0.706 – 1.172 | 0.664 – 1.437 | 0.275 – 1.078 | 0.679 – 1.316 |  |
| Length-of-stay | 0.759 – 1.173 | 0.527 – 1.018 | 0.532 – 1.476 | 0.593 – 1.060 |  |

1. **All** **analyses were corrected for age and sex**. All outcomes were analyzed in separate multivariable regression models resulting in odds or hazard ratio for each ABO blood group and RhD status using ABO blood group O and RhD negative as references [↑](#endnote-ref-1)
2. Cox regression [↑](#endnote-ref-2)
3. Days alive and free of invasive ventilation [↑](#endnote-ref-3)
4. Days alive and free of circulatory support [↑](#endnote-ref-4)
5. Maximum Sequential Organ Failure Assessment score during the length of stay [↑](#endnote-ref-5)
6. **All** **analyses were corrected for age and sex**. All outcomes were analyzed in separate multivariable regression models resulting in odds or hazard ratio for each ABO blood group and RhD status using ABO blood group O and RhD negative as references [↑](#endnote-ref-6)
7. Cox regression [↑](#endnote-ref-7)
8. Days alive and free of invasive ventilation [↑](#endnote-ref-8)
9. Days alive and free of circulatory support [↑](#endnote-ref-9)
10. Maximum Sequential Organ Failure Assessment score during the length of stay

    * P < 0.05 [↑](#endnote-ref-10)
11. **All** **analyses were corrected for age and sex**. All outcomes were analyzed in separate multivariable regression models resulting in odds or hazard ratio for each ABO blood group and RhD status using ABO blood group O and RhD negative as references [↑](#endnote-ref-11)
12. Cox regression [↑](#endnote-ref-12)
13. Days alive and free of invasive ventilation [↑](#endnote-ref-13)
14. Days alive and free of circulatory support [↑](#endnote-ref-14)
15. Maximum Sequential Organ Failure Assessment score during the length of stay

    * P < 0.05 [↑](#endnote-ref-15)
16. **All** **analyses were corrected for age and sex**. All outcomes were analyzed in separate multivariable regression models resulting in odds or hazard ratio for each ABO blood group and RhD status using ABO blood group O and RhD negative as references [↑](#endnote-ref-16)
17. Cox regression [↑](#endnote-ref-17)
18. Days alive and free of invasive ventilation [↑](#endnote-ref-18)
19. Days alive and free of circulatory support [↑](#endnote-ref-19)
20. Maximum of Sequential Organ Failure Assessment score during the length of stay [↑](#endnote-ref-20)
21. **All** **analyses were corrected for age and sex**. All outcomes were analyzed in separate multivariable regression models resulting in odds or hazard ratio for each ABO blood group and RhD status using ABO blood group O and RhD negative as references [↑](#endnote-ref-21)
22. Cox regression [↑](#endnote-ref-22)
23. Days alive and free of invasive ventilation [↑](#endnote-ref-23)
24. Days alive and free of circulatory support [↑](#endnote-ref-24)
25. Maximum of Sequential Organ Failure Assessment score during the length of stay [↑](#endnote-ref-25)
