# Supplementary material for: ABO and RhD blood group are not associated with mortality and morbidity in critically ill patients; a multicentre observational study of 29 512 patients
Source: BMC Anesthesiol. 2022 Apr 2;22:91. doi: 10.1186/s12871-022-01626-4 (PMC8976170; doi:10.1186/s12871-022-01626-4)
Supplement: Supplementary file 2 — Additional file 2. [file 12871_2022_1626_MOESM2_ESM.docx]

|  | | | | | | | | |
| --- | --- | --- | --- | --- | --- | --- | --- | --- |
| **Additional file 2. Distribution of ABO and RhD blood groups in patients with/without COVID-19** | | | | | |  |  |  |
| **COVID-19** | **YES** | **NO** | | **P-value^[[1]](#endnote-1)^** | | | |  |
| **N** | **338** | **29 174** | |  | | | |  |
| **Blgrp A, %** | 41 | 44 | | 0.293 | | | |  |
| **Blgrp B, %** | 13 | 12 | | 0.320 | | | |  |
| **Blgrp AB, %** | 9.5 | 4.7 | | <0.001 | | | |  |
| **Blgrp O, %** | 36 | 40 | | 0.177 | | | |  |
|  |  | |  | | | |  |  |
| **RhD positive, %** | 87 | 84 | | | 0.100 | | |  |
| **RhD negative, %** | 13 | 16 | | | 0.100 | | |  |

1. Chi-square test [↑](#endnote-ref-1)
